# Comparison and reproducibility of standard and high temporal resolution myocardial tissue tagging in patients with severe aortic stenosis

**DOI:** 10.1186/1532-429X-13-S1-P311

**Published:** 2011-02-02

**Authors:** Christopher D Steadman, Naveed A Razvi, Kym IE Snell, Joost PA Kuijer, Albert C van Rossum, Gerry P McCann

**Affiliations:** 1 Department of Cardiovascular Sciences, University of Leicester, Leicester, UK; 2 NIHR Leicester Cardiovascular Biomedical Research Unit, Leicester, UK; 3 Department of Physics and Medical Technology, ICaR-VU, VU University Medical Center, Amsterdam, Netherlands; 4 Department of Cardiology, ICaR-VU, VU University Medical Center, Amsterdam, Netherlands

## Objectives

The aim of this study was to compare and assess the reproducibility of left ventricular (LV) circumferential peak systolic strain (PeakE_cc_) and strain rate (SR) measurements using standard and high temporal resolution myocardial tissue tagging in patients with severe aortic stenosis (AS).

## Background

Myocardial tissue tagging with cardiac magnetic resonance (CMR) can be used to quantify strain and SR, however, there are little data on the reproducibility. Diastolic SR may be of particular interest as it may be the most sensitive marker of diastolic dysfunction often occurring early in the course of disease.

## Methods

Eight patients with isolated severe AS without obstructive coronary artery disease were prospectively enrolled. They underwent CMR in a 1.5T scanner (Siemens Avanto) on two separate occasions, median interval 12 days. Complementary tagged (CSPAMM) images were acquired with both a single breath-hold (SBH: temporal resolution 42ms), and a multiple brief expiration breath-hold (MBH: high temporal resolution 17ms) sequence. Mid-wall PeakE_cc_ was measured in the LV at mid-ventricular level with HARP Version 2.7 (Diagnosoft, USA). SR was calculated from the strain data; SR=E_cc_2-E_cc_1/Time2-Time1. PeakE_cc_ , peak systolic and diastolic SR were read from curves of strain and SR against time. The MBH SR curves were filtered with a moving average (MA) to reduce noise sensitivity, results from a sample width of three and five were examined. Differences between SBH and MBH were assessed using Wilcoxon signed-rank test as not all measures were normally distributed. Reproducibility assessments were carried out on all techniques.

## Results

**Figure 1:**
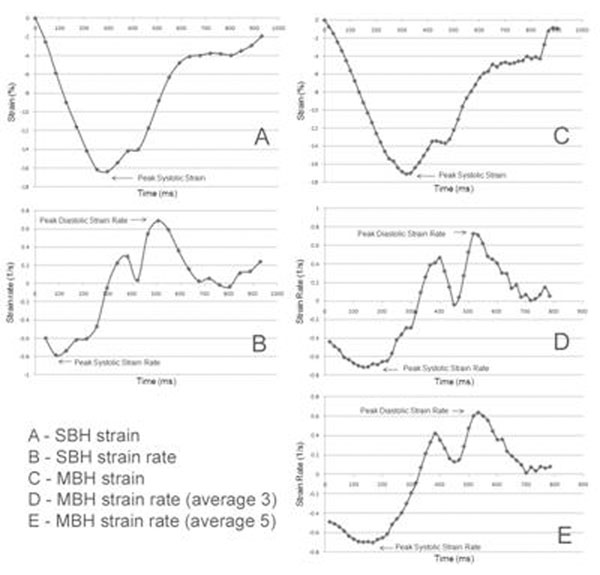
Strain and SR curves

PeakE_cc_ was significantly higher with MBH vs. SBH, but reproducibility was slightly worse. Results are summarised in Table 1. Systolic SR was approximately equal with all techniques although MBH using MA of five led to a borderline significant reduction. Diastolic SR was higher when measured with MBH although only significant using MA of three. Systolic and diastolic SR measures were more reproducible with MBH compared with SBH, except for the diastolic SR using MA of three, which was substantially worse. Strain and SR curves for the same patient are shown in Figure 1.

**Table 1:** Results

		Peak systolic strain (%)	Peak systolic strain rate (1/s)	Peak diastolic strain rate (1/s)
SBH		-13.7Â±2.4	-0.74Â±0.15	0.75Â±0.27
MBH (MA of three)		-15.1Â±3.1 (p=0.023 vs. SBH)	-0.73Â±0.11 (p=0.877 vs. SBH)	1.12Â±0.54 (p=0.017 vs. SBH)
MBH (MA of five)			-0.69Â±0.10 (p=0.049 vs. SBH)	0.91Â±0.36 (p=0.535 vs. SBH)
SBH reproducibility	MDÂ±SD CoV B-A		0.50Â±1.52 11.1% -2.5 to 3.5	-0.01Â±0.13 18.1% -0.26 to 0.28	-0.04Â±0.16 21.0% -0.36 to 0.27
MBH reproducibility (MA of three)	MDÂ±SD CoV B-A	1.13Â±2.23 14.7% -3.3 to 5.6	0.06Â±0.04 5.3% -0.02 to 0.14	-0.13Â±0.44 39.0% -1.00 to 0.75
MBH reproducibility (MA of five)	MDÂ±SD CoV B-A		0.04Â±0.05 7.8% -0.07 to 0.15	0.09Â±0.15 16.9% -0.39 to 0.22

MD±SD=mean difference ± standard deviation, CoV=coefficient of variation, B-A=Bland-Altman 95% limits of agreement

## Conclusions

It is likely than SBH may be adequate or even superior to MBH for assessment of PeakE_cc_. The increased temporal resolution of MBH may be advantageous for examining systolic and diastolic SR; a MA of five for diastolic SR may be the preferred method for quantification given the improved reproducibility of this measure.

